# Outcomes and Prognosis of COVID-19-Induced Adult Respiratory Distress Syndrome Patients Treated with Prolonged Veno-Venous Extracorporeal Membrane Oxygenation: A Retrospective Multicenter Study

**DOI:** 10.3390/jcm13237252

**Published:** 2024-11-28

**Authors:** Amram Bitan, Nitzan Sagie, Eduard Ilgiyaev, Dekel Stavi, Maged Makhoul, Arie Soroksky, Yigal Kasif, Victor Novack, Ori Galante

**Affiliations:** 1 Medical Division, Soroka University Medical Center, Faculty of Health Sciences, Ben Gurion University of the Negev, Beer-Sheva 84101, Israel; amrambi@clalit.org.il; 2 Clinical Research Center, Soroka University Medical Center, Faculty of Health Sciences, Ben Gurion University of the Negev, Beer-Sheva 8410501, Israel; sagieni@post.bgu.ac.il (N.S.); victorno@clalit.org.il (V.N.); 3 Shamir Medical Center, Faculty of Medicine, University of Tel Aviv, Be’er Ya’akov 703301, Israel; adii@shamir.gov.il; 4 Division of Anesthesia, Pain Management, and Intensive Care, Tel-Aviv Sourasky Medical Center—Ichilov Hospital, Faculty of Medicine, University of Tel Aviv, Tel Aviv 6423906, Israel; dekelst@tlvmc.gov.il; 5 Department of Cardiac Surgery, Rambam Health Care Campus, Haifa 3109601, Israel; m_makhoul@rambam.health.gov.il; 6 Intensive Care Unit, Wolfson Medical Center, Faculty of Medicine, University of Tel Aviv, Holon 5822012, Israel; aries@wmc.gov.il; 7 Department of Cardiac Surgery, Tel HaShomer Hospital, Ramat Gan 5262000, Israel; yigal_k@bbalev.co.il; 8 Medical Intensive Care Unit, Soroka University Medical Center, Faculty of Health Sciences, Ben Gurion University of the Negev, Beer-Sheva 84101, Israel

**Keywords:** prolonged ECMO, COVID-19, ARDS

## Abstract

**Background:** Predicting whether extracorporeal membrane oxygenation (ECMO) treatment duration affects prognosis is important both medically and economically. **Methods**: We conducted a retrospective, multicenter study to better understand the outcomes of patients treated with veno-venous (VV) ECMO over a prolonged duration, analyzing data from the Israel ECMO registry. The study included all adult patients treated with VV-ECMO due to COVID-19-induced respiratory failure. The primary outcomes were survival rates up to 180 days from cannulation. **Results:** One hundred and eighty-eight patients were included in the study. The median age was 50 years (IQR 42, 50), and 69% were male. Patients were mechanically ventilated for a median of 2.5 days before cannulation (IQR 0.5, 5). The mean ECMO support duration was 29.9 days, with a maximal duration of 189.9 days. The survival rate for 180 days was 56%. We found no change in survival for patients on ECMO for 14, 28, or 56 days. Every day of mechanical ventilation before cannulation correlated with an 11% greater risk for prolonged ECMO treatment (*p* = 0.01). **Conclusions:** COVID-19-induced ARDS patients treated with VV-ECMO for prolonged duration had the same prognosis as those treated for short periods of time. The longer the duration of mechanical ventilation before ECMO cannulation, the higher the risk for prolonged ECMO treatment.

## 1. Introduction

The use of veno-venous (VV) extracorporeal membrane oxygenation (ECMO) treatment for respiratory failure has increased exponentially over the last decades [1]. Until 2009, evidence regarding survival rates for VV-ECMO patients was unfavorable, with a relatively low number of ECMO cases reported worldwide [2,3]. The CESAR trial, with data gathered during the H1N1 and more recently during COVID-19 pandemics, highlighted survival benefits for patients treated with VV-ECMO for severe Adult Respiratory Distress Syndrome (ARDS) [4,5,6]. Since 2009, the number of patients treated annually with VV-ECMO has increased to 4000 ECMO cases [7]. According to the extracorporeal life support organization (ELSO) registry, along with the rising number of ECMO cases, the mean ECMO treatment duration has also increased from a median of 7 days during 2000 and 2012 to a mean of 12.5 days in 2016 [3,8]. In 2018, 20% of all ECMO runs were defined as prolonged (longer than 14 days) [1].

Until the COVID-19 pandemic, ECMO support lasting less than 14 days was considered short, while ECMO treatment lasting longer than 14 days was deemed prolonged [1], though some papers suggested 21 days [9] or even 28 days [10] as a definition for prolonged ECMO. We aimed to study the outcomes and risk factors of this poorly defined and understudied group of patients who were treated with VV-ECMO for prolonged duration. Whether ECMO duration affects prognosis and whether certain patient characteristics, lab results, or treatment-related parameters can predict treatment duration remains unknown. Yet, these questions are of extreme medical and economic significance, as ECMO is a highly resource-demanding treatment.

## 2. Methods

We conducted a retrospective, multi-center study based on data collected from the Israeli ECMO registry (originated in 2019).

We extracted data on all adult patients (>18 years old) who had been treated with VV-ECMO due to PCR-positive COVID-19-induced ARDS at six ECMO centers. Patients’ records with poor data collection were excluded.

The data collected included the following: (1) demographic information, comorbidities, and pre-ECMO baseline parameters such as respiratory support modality, prone position, gas exchange data, duration of invasive mechanical ventilation before ECMO cannulation, and treatment with nitric oxide, inotropes, or vasopressors; (2) ECMO-related parameters such as total ECMO duration, maximal blood flow, and maximal gas flow; and (3) ECMO and non-ECMO-related complications such as thrombotic events (e.g., venous or arterial thrombosis, heparin-induced thrombocytopenia, and ECMO circuit thrombosis necessitating circuit replacement), bleeding defined as requiring treatment with blood products, and infections, including ventilator-associated pneumonia and bacteremia.

The primary outcomes were 180-day mortality and ECMO treatment duration. The secondary outcomes were complication rates, parameters that predict ECMO treatment duration, and prognosis in prolonged ECMO patients.

This study was conducted according to the amended Declaration of Helsinki (2024). Each medical center’s ethical committee approved the study.

### Statistical Analysis

All statistical analyses were carried out using the R software, version 4.3.1 (R Foundation for Statistical Computing, Vienna, Austria).

Numerical variables are summarized using medians and interquartile ranges (IQRs), while categorical data, including binary variables, such as comorbidities, are presented as percentages. Patients are classified based on their ECMO status (connected/disconnected) at specific time points during follow-up; therefore, some patients are included in all groups. A quasi-Poisson regression model was employed to assess the association between pre-ECMO predictors and outcomes. The outcome variable encompassed death or continued ECMO utilization within an 8-week (56 days) period. To investigate whether certain predictors were linked to mortality, Cox regression was performed for specific study groups. Patient survival is presented using Kaplan–Meier plots, and comparisons of Kaplan–Meier rates between different groups were performed using a bootstrapping analysis. This method involves a resampling technique that generates multiple replications of the original dataset through random sampling with replacement. This approach enables the estimation of sampling variability, facilitating the assessment of significant differences in survival rates amongst non-mutually exclusive groups. Bootstrapping was employed to determine whether a difference in the Kaplan–Meier survival rates significantly deviated from zero. Statistical significance was ascertained using a *p*-value threshold of <0.05.

## 3. Results

Out of 193 records, 188 from six ECMO centers were included in the study, and only five patients were excluded due to missing essential data. Demographics and medical data are shown in Table 1. Patients’ median age was 50 (IQR 42, 50) years, 69% were male, and the median body mass index (BMI) was 31 kg/m^2^ (IQR 27, 35). Diabetes and cardiovascular disease were the most common comorbidities (22% and 8.5% of patients, respectively). The median sequential organ failure assessment (SOFA) score on admission was 8 (IQR 6, 11), and 86% were mechanically ventilated before cannulation, with a median of 2.5 days (IQR 0.5, 5) on mechanical ventilation before cannulation. A total of 53% of the patients were treated with nitric oxide, 34% were proned, and 18% required inotrope support before cannulation. The mean ECMO support duration was 29.9 days (IQR 8, 36.7) with a maximal duration of 189.9 days. Information on survival data was available for 183 patients.

**Figure 1:**
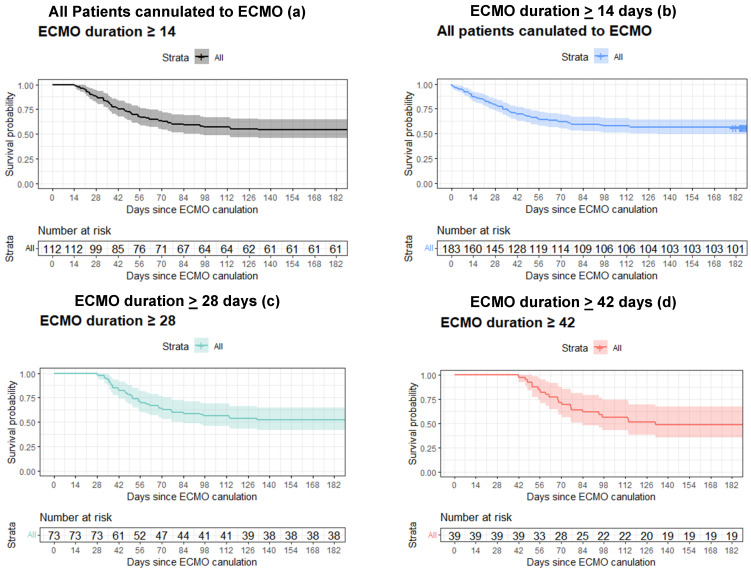

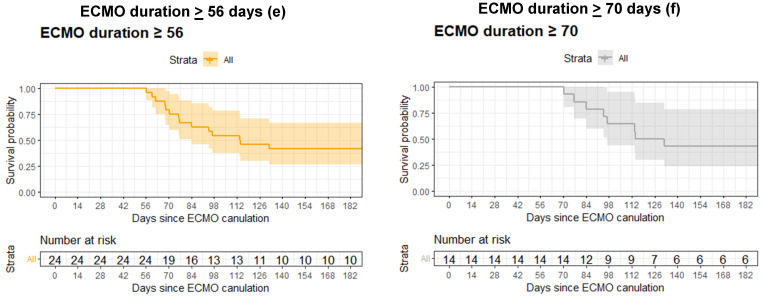
Kaplan–Meier plots describing the 6-month survival rates of COVID-19 ARDS patients divided by their time on ECMO. (**a**) All patients; (**b**) patients still on ECMO at day 14 from cannulation; (**c**) patients still on ECMO at day 28 from cannulation; (**d**) patients still on ECMO at day 42 from cannulation; (**e**) patients still on ECMO on day 56 from cannulation; and (**f**) patients still on ECMO on day 70 from cannulation. Extracorporal membrane oxygenation (ECMO).

Figure 1 describes the survival rate of patients divided by their time on ECMO. Each plot shows survival rates of up to 180 days from cannulation for patients who were still alive and on ECMO (i.e., not deceased or decannulated) at 0, 14, 28, 42, 56, and 70 days from cannulation. These plots are designed to resemble prospectivity by representing accumulative survival data up to specific days, aiming to aid clinicians in assessing their patients’ outcomes at these different time points.

The overall patients’ survival rates did not change significantly up to 180 days. In a subgroup analysis of patients still on ECMO on day 56, there was a non-significant trend toward a reduced survival rate when comparing survival at 90 and 180 days.

Table 2 describes survival rates for 60, 90, and 180 days for all ECMO patients and for patients still on ECMO at 14, 28, 42, and 56 days (i.e., did not die or undergo decannulation). The overall survival rates for 60, 90, and 180 days were 63%, 59%, and 56%, respectively. There was no significant difference in survival for patients who were still alive and on ECMO amongst the different time groups.

We sought to assess different risk factors for mortality and prolonged the ECMO treatment. Table 3 shows a regression model assessing the correlation between several parameters (duration of mechanical ventilation before ECMO cannulation, SOFA score, prone position, and treatment with nitric oxide, vasopressors, or inotropes) and prolonged ECMO treatment. Outcomes were adjusted according to age, gender, and BMI. As shown, every day of mechanical ventilation before cannulation added an 11% increased risk for prolonged ECMO treatment (*p* = 0.01).

Table 4 shows univariant analyses assessing different parameters and their correlations with mortality. Only age and SOFA score on the day of cannulation were found to correlate significantly with mortality. The risk for mortality was higher by 3% (*p* = 0.001) for every additional year of age (between 20 and 74 years old) and by 12% (*p* = 0.006) for each point increase in SOFA score on the day of cannulation.

In addition, patients older than 50 years on the day of cannulation had a higher mortality rate (54% vs. 36%, respectively, *p* = 0.013).

Table 5 summarizes the incidence of complications and mortality associated with each complication during ECMO treatment. The most abundant complications were infections, bleeding events, and ECMO-related mechanical complications.

Table 6 shows the correlation between specific complications and mortality, with no complications found to correlate significantly to mortality.

## 4. Discussion

In this retrospective, multicenter study, we aimed to answer two critical questions: (1) Does ECMO treatment duration affect prognosis? and (2) Are there predicting factors for prolonged ECMO duration? These questions are important both medically and economically, as a clinician treating a patient on ECMO for a prolonged time frequently contemplates whether the immense efforts invested in these patients will be worthwhile. Questions on when to discuss lung transplantation or end-of-life treatment frequently arise.

We found no correlation between ECMO duration and survival rates. As shown in Figure 1 and Table 2, 90-day survival for patients on the day of cannulation and for patients still on ECMO at 14, 28, 42, and 56 days were 58%, 59%, 59%, 62%, and 62%, respectively (*p* = 0.75). The survival rate for 180 days also remained relatively steady throughout the treatment period.

In other words, a patient who is still cannulated on days 14, 28, or 56 has the same chance for survival as they had on the day of cannulation. Hence, we suggest that ECMO treatment duration does not affect prognosis and, therefore, should not be considered as a factor when contemplating issues of level of care and end-of-life treatment.

Several previous studies have reported prolonged ECMO outcomes. Flinspach et al. [11] studied a group of 117 patients in a single center, reporting their outcomes at four different times: up to 14, 14–28, 29–50, and above 50 days. Despite an overall relatively low survival of 35%, they did not find a statistically significant difference in mortality rates amongst the different time groups and concluded that ECMO duration did not increase mortality.

Stern et al. [12] studied a smaller group of 44 ECMO patients, divided between <90 days of ECMO treatment and >90 days, who had remarkable survival rates of overall 82% to hospital discharge. They found a significantly higher mortality in patients treated with ECMO for more than 90 days (62% vs. 90%, respectively). It may be argued that these exceptional survival rates at this single-center study may be due to high selectivity, which is also suggested by their young mean patient age of 40 years, as compared to 50 and 54 years in the current study and Flinspach et al. [11].

In a recent retrospective large-scale study based on the ELSO registry, Abhimanyu et al. analyzed data from 13,681 patients treated with VV-ECMO for ARDS [13]. They found that the duration of VV-ECMO (per additional day) was significantly associated with reduced survival to hospital discharge in patients supported with VV-ECMO for <21 days. However, when the analysis was restricted to patients supported with VV-ECMO for ≥21 days, duration was not significantly associated with mortality.

In a subgroup analysis of 24 patients still cannulated at 56 days, we found a non-statistically significant trend toward a reduction in the survival rate after 90 days from cannulation. Their 90-day survival rate was 62%, which declined to 41% at 180 days (*p* = 0.13). This finding may suggest that days 60–90 on ECMO may serve as a landmark for prognostic evaluation (i.e., if the patient is not improving and there are no signs of lung recovery during the 3rd month on ECMO, the possibility of lung transplantation should be discussed). In our cohort, 3/24 (12.5%) patients who were still on ECMO on day 56 underwent lung transplantation after 121, 144, and 189 days, 2 of whom survived to 6 months. The assumption of the 3rd month of ECMO being an important landmark for prognostic evaluation is supported by Levi et al., who reported on an Israeli series of 20 COVID-19 patients treated with ECMO and listed for lung transplantation. The median duration from hospitalization to listing was 85.5 days. The median duration on the waitlist was 25.5 days. A total of 4 patients underwent lung transplantation, while 9 out of the remaining 16 recovered without transplantation after a median of 59 days on ECMO. Seven patients died while waiting for lung transplantation after a median of 101 days on ECMO [14].

We found time on mechanical ventilation before ECMO cannulation to be the only significant risk factor for prolonged ECMO (>56 days), with an 11% higher risk for prolonged ECMO duration for every day of invasive ventilation before ECMO cannulation. These findings support the hypothesis that the sooner VV-ECMO is initiated, the better, as supported by Abhimanyu et al., who also found that longer duration of mechanical ventilation before VV-ECMO (81 vs. 49 h, respectively) was associated with prolonged ECMO duration [13].

Time on mechanical ventilation before ECMO cannulation is a well-known prognostic factor for VV-ECMO patients [12,15,16,17]. In the current study, we did not find a significant correlation between duration of ventilation before ECMO and mortality. We assume a possible explanation might be that in our cohort there was a relatively short mean duration between invasive ventilation and ECMO cannulation, a mean of 2.5 days (IQR 0.5–5) as compared to 4 or 7 days in previous studies [15,16,17].

We found that older age and higher SOFA score increased the risk for mortality: 3% for every year (between the ages of 20 and 74 years) and 12% for every point of SOFA score. Age older than 50 years was also found to correlate significantly with mortality.

Stern et al. [12] found age and SOFA scores to be significantly higher in patients who required prolonged ECMO duration, but these factors did not correlate with higher mortality rates.

A high-quality systematic review, including over 17,000 patients [15], also found age to be a statistically significant prognostic factor.

ECMO is a highly invasive treatment that exposes patients to adverse events such as infections, bleeding, and ECMO-related mechanical complications. We found an incidence of approximately 30% for bleeding and 44% for infections. Yet, none of these complications correlated significantly to mortality in our study. In a meta-analysis of 4800 patients [18], a similar pneumonia rate of 29–38% was found among COVID-19 ECMO patients. The bacteremia rate was 12–17%, which is lower than in the current study (38%).

The Israeli ECMO registry lacks the timing for every complication, so we could not assess causality or connection between complications and prolonged ECMO duration. Nonetheless, it is reasonable that the longer the exposure to ECMO, the higher the probability for certain ECMO-related complications to occur, such as mechanical complications, bleeding, and infections. Stern et al. [12] found that bleeding and infections were more common after >90 days on ECMO (76.9% vs. 58.1% for bleeding (*p* = 0.31), 84% vs. 51% for pneumonia (*p* = 0.04), and 84% vs. 48% for bacteremia (*p* = 0.026), respectively). We found no correlation between complications and mortality; hence, we suggest that a high incidence of complications should not be regarded as a factor when discussing the level of care or trying to assess prognosis or treatment failure.

Our study is not without limitations. Being a retrospective registry-based study, selection bias is probable. We studied only COVID-19-related ARDS patients, so our results cannot be generalized to all VV-ECMO patients, though the comparable results reported by Abhimanyu et al. [13], who studied all ARDS VV-ECMO patients, suggest generalizability.

## 5. Conclusions

In this retrospective multicenter study, we found no correlation between the duration of ECMO treatments and the prognosis of patients with COVID-19-induced ARDS. ECMO duration should not be considered as a single factor when contemplating issues of level of care and end-of-life treatment, as it does not predict treatment failure or higher mortality rates.

## Figures and Tables

**Table 1:** Baseline characteristics of patients treated with VV-ECMO for COVID-19 ARDS.

Demographics	*N* = 188
Age on admission in years, median (IQR)	50 (42, 58)
Gender (Male), *N* (%)	129 (69%)
BMI, kg/m^2^ median (IQR)	31 (27, 35)
**Medical history**	
Smoking	11 (5.9%)
COPD	4 (2.1%)
Diabetes	42 (22%)
Chronic Renal disease	5 (2.7%)
Cardiovascular disease	16 (8.5%)
SOFA score on admission, median (IQR)	8.00 (6.00, 11.00)
PRE-ECMO conventional mechanical ventilation	162 (86.1%)
Duration of PRE-ECMO invasive mechanical ventilation (days), median (IQR)	2.5 (0.5, 5.0)
PRE-ECMO prone positioning	63 (34%)
PRE-ECMO inotropic or vasoactive drugs	33 (18%)
PRE-ECMO nitric oxide	100 (53%)

Abbreviations: extracorporeal membrane oxygenation (ECMO); interquartile range (IQR); sequential organ failure assessment (SOFA); body mass index (BMI); chronic obstructive pulmonary disease (COPD).

**Table 2:** Survival rates of COVID-19-induced ARDS ECMO patients since cannulation by treatment landmarks.

	All Patients N = 183	Patients Still on ECMO on Day 14 N = 112	*p*-Value *	Patients Still on ECMO on Day 28 N = 73	*p*-Value *	Patients Still on ECMO on Day 42 N = 39	*p*-Value *	Patients Still on ECMO on Day 56 N = 24	*p*-Value *
60 days survival Rate (95% CI)	0.63 (0.57–0.71)	0.66 (0.58–0.75)	0.636	0.68 (0.58–0.70)	0.440	0.79 (0.68–0.93)	0.036	---	---
90 days survival Rate (95% CI)	0.58 (0.52–0.66)	0.59 (0.50–0.69)	0.916	0.59 (0.48–0.71)	0.988	0.62 (0.48–0.79)	0.732	0.62 (0.45–0.85)	0.756
180 days survival Rate (95% CI)	0.56 (0.49–0.63)	0.54 (0.46–0.65)	0.840	0.52 (0.41–0.64)	0.496	0.49 (0.35–0.67)	0.468	0.41 (0.25–0.66)	0.132

* The *p*-value was calculated using bootstrapping analysis with the null hypothesis, assuming no difference in the Kaplan–Meier rate. Abbreviations: confidence Interval (CI); extracorporeal membrane oxygenation (ECMO).

**Table 3:** Regression models predicting factors for prolonged ECMO duration beyond 56 days from cannulation.

	ECMO Duration > 56 Days		
Factor	IRR	95% CI	*p*-Value
PRE-ECMO mechanical ventilation (days)	1.11	0.98, 1.09	0.01
SOFA score	0.90	0.78, 1.03	0.13
PRE-ECMO prone positioning	1.53	0.69, 3.31	0.29
PRE-ECMO nitric oxide	0.74	0.29, 1.88	0.52
PRE-ECMO inotropic vasoactive drugs	1.24	0.31, 3.64	0.73

Abbreviations: confidence Interval (CI); extracorporeal membrane oxygenation (ECMO); incidence rate ratio (IRR); sequential organ failure assessment (SOFA).

**Table 4:** Results of univariate COX regressions between demographics, comorbidities, and pre-ECMO parameters to mortality.

Characteristic	All Patients Cannulated to ECMO, N = 183		
	HR	95% CI	*p*-Value
Gender (male)	1.61	0.96, 2.70	0.069
Age on admission (years)	1.03	1.01, 1.05	0.001
BMI (kg/m^2)^	1.00	0.96, 1.03	0.816
SOFA score on the day of cannulation	1.12	1.03, 1.22	0.006
COPD	0.45	0.06, 3.22	0.425
Diabetes	0.90	0.53, 1.55	0.714
Cardiovascular disease	1.46	0.73, 2.93	0.283
Smoking	1.45	0.63, 3.34	0.379
Days between Symptoms and intubation	1.00	1.00, 1.01	0.364
Days on invasive mechanical ventilation before ECMO cannulation	1.01	0.97, 1.05	0.602
PRE-ECMO-prone positioning	0.96	0.60, 1.52	0.851
PRE-ECMO conventional mechanical ventilation	1.03	0.63, 1.66	0.920
PRE-ECMO inotropic vasoactive drugs	0.76	0.41, 1.41	0.383
PRE-ECMO nitric oxide	1.16	0.75, 1.81	0.506

Abbreviations: body mass index (BMI); extracorporeal membrane oxygenation (ECMO); hazard ratio (HR); confidence Interval (CI); chronic obstructive pulmonary disease (COPD); sequential organ failure assessment (SOFA).

**Table 5:** Clinical complications during ECMO treatment.

Complications		Number of Patients (N = 188)	Mortality Rates
Renal	Need for renal replacement therapy	26 (13%)	61%
CNS	Convulsions	5 (2.7%)	60%
	Brain infarct	4 (2.1%)	25%
	Brain edema diffuse ischemia	1 (0.5%)	100%
Infections	Bacteremia sepsis	61 (32%)	38.3%
	Ventilation-associated pneumonia	50 (27%)	24.5%
Mechanical	Cannula repositioning and reinsertion	17 (9%)	25%
	Entire circuit replacement	29 (15%)	25%
	Tubing or oxygenator clotting requiring replacement	24 (13%)	30.4%
	Pump failure or breakage	3 (1.6%)	66.6%
Bleeding	Number of blood products (mean (range))	9 (0–269)	N/A
	GI bleeding	14 (7.4%)	53.8%
	Peripheral cannulation and or indwelling lines site bleeding	25 (13%)	37.5%
	Mucosal bleeding mouth or nose	25 (13%)	32%
	Thrombocytopenia HIT	16 (8.5%)	25%
	Hemothorax on ECMO	6 (3.2%)	66.7%
Pulmonary	Pneumothorax on ECMO	15 (8%)	40%

Abbreviations: central nervous system (CNS); extracorporeal membrane oxygenation (ECMO); gastrointestinal (GI); heparin-induced thrombocytopenia (HIT).

**Table 6:** Association between ECMO complications and 180-day mortality.

Characteristic	N	OR	95% CI	*p*-Value
Convulsions	188	5.04	0.73, 99.6	0.2
Bacteremia	188	1.04	0.56, 1.92	0.9
Ventilator-associated pneumonia	188	0.47	0.23, 0.92	0.030
Urinary tract infection	188	0.33	0.05, 1.41	0.2
Cannula dislodgment requiring repositioning and reinsertion	188	0.64	0.21, 1.75	0.4
Entire circuit replacement	188	0.59	0.25, 1.32	0.2
Tubing or oxygenator clotting requiring replacement	188	0.56	0.22, 1.36	0.2
Pump failure or breakage	188	2.46	0.23, 53.4	0.5
Number of Blood products	188	1.00	1.00, 1.01	0.4
GI bleeding	188	1.68	0.56, 5.30	0.4
Peripheral cannulation and or indwelling lines site bleeding	188	0.94	0.40, 2.20	0.9
Mucosal bleeding mouth nose	188	0.94	0.40, 2.20	0.9
Heparin Induced Thrombocytopenia (HIT)	188	0.71	0.23, 1.99	0.5
Hemothorax on ECMO	188	6.37	1.00, 123	0.094
Pneumothorax on ECMO	188	0.79	0.26, 2.30	0.7

Abbreviations: odds ratio (OR); confidence interval (CI); gastrointestinal (GI); heparin-induced thrombocytopenia (HIT).

## Data Availability

The datasets used and/or analyzed during the current study are available from the corresponding author upon reasonable request.

## Abbreviations

ARDS	Adult Respiratory Distress Syndrome
BMI	Body Mass Index
CNS	Central Nervous System
COPD	Chronic Obstructive Pulmonary Disease
CL	Confidence Limit
ECMO	Extracorporeal Membrane Oxygenation
ELSO	Extracorporeal Life Support Organization
GI	Gastrointestinal
HIT	Thrombocytopenia
HR	Hazard Ratio
IQR	Interquartile Range
OR	Odds Ratio
SOFA	Sequential Organ Failure Assessment
VV	Veno-Venous
